# Hepatic arterial infusion chemotherapy, lenvatinib plus programmed cell death protein‐1 inhibitors: A promising treatment approach for high‐burden hepatocellular carcinoma

**DOI:** 10.1002/cam4.7105

**Published:** 2024-04-30

**Authors:** Shumin Fu, Yongkang Xu, Ye Mao, Mengting He, Zhimeng Chen, Shenglan Huang, Dan Li, Yaqin Lv, Jianbing Wu

**Affiliations:** ^1^ Department of Oncology, The Second Affiliated Hospital, Jiangxi Medical College Nanchang University Nanchang Jixangxi China; ^2^ Department of Hepatobiliary and Pancreatic Surgery, The Second Affiliated Hospital, Jiangxi Medical College Nanchang University Nanchang Jixangxi China

**Keywords:** hepatic arterial infusion chemotherapy, hepatocellular carcinoma, lenvatinib, programmed cell death protein‐1 (PD‐1) inhibitors

## Abstract

**Background:**

Hepatic arterial infusion chemotherapy (HAIC) has demonstrated remarkable local therapeutic efficacy in treating patients with large unresectable hepatocellular carcinoma (HCC). Additionally, the combination of lenvatinib and programmed cell death protein‐1 (PD‐1) inhibitors has demonstrated promising antitumor effects in unresectable HCC. Therefore, we conducted a retrospective analysis to evaluate the efficacy and safety of combining HAIC with lenvatinib and PD‐1 inhibitors as a first‐line therapeutic approach in high‐burden HCC patients.

**Methods:**

We conducted a retrospective analysis on patients diagnosed with high‐burden HCC who had major portal vein tumor thrombosis (Vp3 and Vp4) or tumor occupancy exceeding 50% of the liver. These patients received a first‐line treatment consisting of HAIC with a combination of 5‐fluorouracil, leucovorin, and oxaliplatin (FOLFOX), along with lenvatinib and PD‐1 inhibitors between November 2020 and June 2023. The primary endpoints of this study included progression‐free survival (PFS) and overall survival (OS), while the secondary endpoints were objective response rate (ORR), disease control rate (DCR), and treatment‐related adverse events (TRAEs).

**Results:**

Ninety‐one patients were enrolled in this study, with a median PFS of 8.8 months (95% confidence interval [CI]: 5.75–11.78) and a median OS of 14.3 months (95% CI: 11.23–17.31). According to RECIST 1.1 criteria, the ORR was 52.7%, and DCR was 95.6%. According to the mRECIST criteria, the ORR was 72.5%, and the DCR was 96.5%. Among all patients, 86 (94.5%) experienced TRAEs, and there were no instances of treatment‐related deaths.

**Conclusion:**

The combination of HAIC‐FOLFOX with lenvatinib and PD‐1 inhibitors as a first‐line therapy has exhibited notable therapeutic efficacy and well‐tolerated adverse events among patients with high‐burden HCC.

## 1 INTRODUCTION

Primary liver cancer ranked as the sixth most prevalent cancer and the third leading cause of cancer‐related deaths globally in 2020, registering approximately 906,000 novel diagnoses and 830,000 fatalities.^1^ Hepatocellular carcinoma (HCC) accounted for 75%–85% of all primary liver cancer cases.^1^ Despite the continuous improvement in liver cancer surveillance programs, the majority of patients are still diagnosed at advanced stages, rendering them ineligible for curative therapies.^2^ In a groundbreaking study, atezolizumab plus bevacizumab significantly extended the median overall survival (OS) among patients with unresectable HCC while maintaining a manageable toxicity profile.^3^ However, the prognosis for patients with high‐burden features, including first branch portal vein invasion (Vp3), main trunk portal vein invasion (Vp4), and tumor infiltration of ≥50% of the liver volume, remains extremely poor, with a median overall survival (OS) of only 5.5–7.6 months after systemic therapy.^4^, ^5^, ^6^, ^7^, ^8^ These patients usually have a high intrahepatic tumor burden, and standard systemic therapies may not yield the desired antitumor response.

Hepatic arterial infusion chemotherapy (HAIC) is a locoregional interventional treatment method that allows for the direct administration of chemotherapeutic agents into tumor‐feeding vessels. This approach theoretically leads to a greater intratumoral concentration of the chemotherapeutic agent, effectively reducing the tumor burden.^9^ Recent research has indicated that HAIC shows promise in terms of clinical efficacy for the treatment of advanced HCC characterized by major portal vein tumor thrombosis (PVTT) invasion (Vp3 and Vp4) and a high tumor burden.^7^, ^10^, ^11^ However, there are currently no widely accepted standard regimens for HAIC in advanced HCC. More recent findings suggest that the use of HAIC with infusional fluorouracil, leucovorin, and oxaliplatin (FOLFOX) regimens, referred to as HAIC‐FOLFOX, provides a significant survival advantage in patients with advanced HCC.^12^

In recent years, significant progress has been made in systematic treatment approaches for advanced HCC, including the use of molecular targeting agents (MTAs) and immune checkpoint inhibitors (ICIs).^13^, ^14^, ^15^, ^16^ Nevertheless, the survival advantage for patients administered MTA or ICI monotherapy remains relatively constrained. The future development direction for advanced HCC lies in combining MTAs with ICIs. A real‐world retrospective study demonstrated the potent antitumor efficacy of combining lenvatinib, a molecular targeted agent, with programmed cell death protein‐1 (PD‐1) inhibitors in advanced HCC.^17^ Furthermore, multiple investigations have suggested that combining HAIC with systemic therapy leads to better outcomes compared to using systemic therapy alone for high‐burden patients with advanced HCC.^5^, ^8^

Hence, our aim was to retrospectively evaluate the effectiveness and safety of combining HAIC‐FOLFOX with lenvatinib and PD‐1 inhibitors as the first‐line treatment for patients with high‐burden HCC.

## 2 METHODS

### 2.1 Patient characteristics

Between November 2020 and June 2023, we screened electronic medical records of patients with advanced HCC who received first‐line treatment with HAIC‐FOLFOX in combination with lenvatinib and PD‐1 inhibitors at the Second Affiliated Hospital of Nanchang University. We included consecutive patients based on the following specific criteria: Patients diagnosed with advanced HCC as per the American Association for the Study of Liver Disease (AASLD) practice guidelines^18^; advanced HCC with tumor involvement of ≥50% of the liver and/or major PVTT, which includes thrombosis in the main trunk (Vp4) and the first‐order branch of the portal vein (Vp3), graded following the 2010 guidelines of the Japan Society of Hepatology^19^; no history of local interventional therapies or systemic therapies; at least one measurable lesion that can be evaluated using both the modified Response Evaluation Criteria in Solid Tumor (mRECIST) and RECIST version 1.1; Child‐ Pugh score ≤7; 18 years or older; and Eastern Cooperative Oncology Group Performance Status (ECOG‐PS) of 0–1. The exclusion criteria included severe cardiovascular, pulmonary, or infectious diseases, as well as the concurrent presence of other malignant tumors. This study received approval from the Ethics Committee of the Second Affiliated Hospital of Nanchang University in China, and informed consent forms were signed by all participants.

### 2.2 Treatments

HAIC employed the Seldinger's technique to puncture the femoral artery and place a catheter into the feeding hepatic artery with the guidance of digital subtraction angiography (DSA). Subsequently, the FOLFOX regimen was administered through the catheter. This regimen included oxaliplatin at a dose of 85 mg/m^2^ infused over 3 h on Day 1, leucovorin at a dose of 400 mg/m^2^ administered from 3 to 5 h on Day 1, and a 5‐fluorouracil bolus at a dose of 400 mg/m^2^ followed by a continuous infusion of 2400 mg/m^2^ over 46 h. Once the arterial chemotherapeutic drug infusion was completed, the catheter and sheath were removed. Lenvatinib and PD‐1 inhibitors were administered within a 5‐day window, either before or after the initiation of HAIC. Lenvatinib was administered orally at an initial dose of 12 mg per day for individuals with a body weight of 60 kg or more, and 8 mg per day for those with a body weight less than 60 kg until disease progression. In our initial exploratory study, we used multiple PD‐1 inhibitors to ensure a larger sample size for investigating the effectiveness of combination immunotherapy. The PD‐1 inhibitors, including tislelizumab (200 mg), sintilimab (200 mg), and camrelizumab (200 mg), were administered intravenously at their standard dosages. HAIC and PD‐1 inhibitors were administered every 3 weeks, with PD‐1 inhibitors continuing every 3 weeks after HAIC was discontinued.

The doses of lenvatinib and arterial chemotherapeutic agents could be adjusted, interrupted, or discontinued in response to treatment‐related adverse events (TRAEs). All patients, including those who underwent liver resection, continued treatment until they experienced disease progression, encountered unacceptable toxicity, or until death occurred. The criteria for liver resection were based on the Chinese expert consensus on neoadjuvant and conversion therapies for hepatocellular carcinoma.^20^ Patients eligible for liver resection met the following criteria: (1) demonstrated a reduction in tumor volume or tumor stage, achieving complete/partial response assessment, or maintaining stable disease for a minimum of 2 months based on mRECIST criteria.; (2) determined by multidisciplinary treatment (MDT) discussion, the tumor was deemed resectable with the possibility of achieving an R0 resection and sufficient residual liver volume and function; (3) had no contraindications for liver resection surgery.

### 2.3 Data collection

We retrieved clinical and radiological data from electronic medical records, including the following information for analysis: age, gender, ECOG PS score, hepatitis etiology, alpha‐fetoprotein (AFP) level, alanine aminotransferase (ALT), aspartate aminotransferase (AST), total bilirubin (TBIL), albumin (ALB), albumin bilirubin (ALBI) grade, maximum tumor size, tumor number, presence or absence of macrovascular invasion, classification of PVTT, and presence or absence of extrahepatic metastasis. All routine laboratory examination data were initially obtained within 1 week before the first treatment and subsequently at intervals of 3 ± 1 weeks during the treatment course. Imaging data, including magnetic resonance imaging (MRI) and contrast‐enhanced computed tomography (CT) scans, were obtained within 1 week before the start of treatment, with subsequent evaluations conducted at intervals of 4–8 weeks.

### 2.4 Outcome assessment

The primary endpoints of this study included progression‐free survival (PFS) and overall survival (OS), while the secondary endpoints encompassed the disease control rate (DCR), objective response rate (ORR), and TRAE. PFS was defined in this study as the time from the initiation of therapy to the first documented disease progression. For patients undergoing surgical resection, even if the entire tumor is completely excised, we opted not to censor these patients. Instead, they continued to be followed up, considering the time of surgery as the endpoint of observation, to ensure a comprehensive assessment of dynamic treatment response. OS was defined as the duration from the initiation of treatment to the time of death from any cause. Tumor response was categorized into complete response (CR), partial response (PR), stable disease (SD), or progressive disease (PD) based on assessments using both mRECIST and RECIST version 1.1 criteria. The ORR was calculated as the proportion of patients achieving a CR plus PR, while the DCR was determined by adding SD to the ORR. TRAEs were assessed following the National Cancer Institute Common Terminology Criteria for Adverse Events (CTCAE) version 5.0.

### 2.5 Statistical analysis

The statistical analyses were conducted using R software version 4.3.1 and IBM SPSS Statistics software version 25.0. Survival curves for PFS and OS were generated utilizing the Kaplan–Meier method. Variables with *p*‐values less than 0.05 were deemed statistically significant in both univariate and multivariate Cox regression analyses.

## 3 RESULTS

**FIGURE 1:**
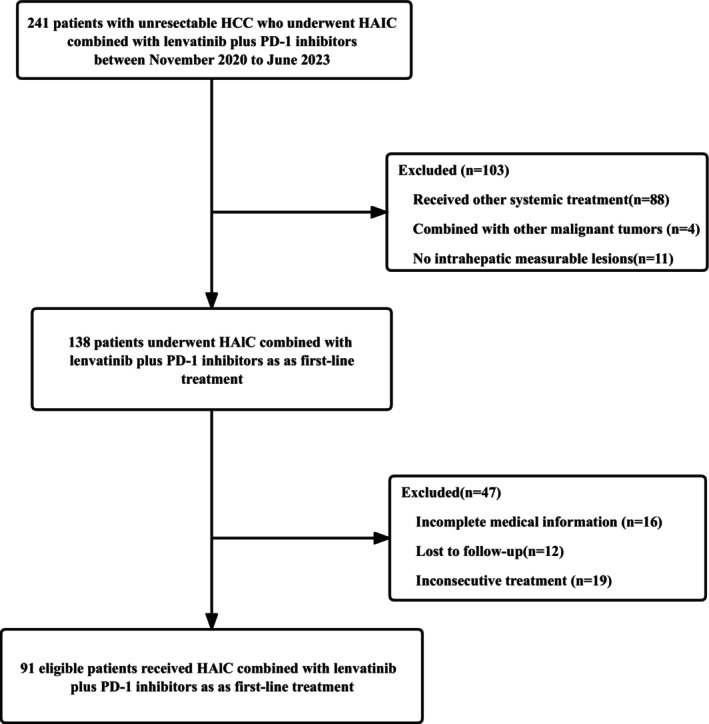
Patient screening process.

Between November 2020 and June 2023, we enrolled a total of 91 high‐burden HCC patients who underwent treatment with HAIC‐FOLFOX in combination with lenvatinib and PD‐1 inhibitors (Figure 1 **)**. Detailed baseline patient characteristics are presented in Table 1. All participants had high‐burden HCC, with 89% of them having major PTVTT (Vp3 or Vp4), 17.6% having extrahepatic metastasis, and 73.6% having a largest tumor diameter greater than 10 cm. The study included predominantly male participants (89%). Among the participants, hepatitis B virus (HBV) infection was the leading cause of HCC (94.5%), and all individuals with HBV‐related HCC received antiviral therapy.

**TABLE 1:** Baseline characteristics of patients.

Characteristic	Number (*n*, %)
Sex	
Male	81 (89.0%)
Female	10 (11.0%)
Age, years	
≥50	50 (54.9%)
<50	41 (45.1%)
ECOG PS	
0	63 (69.2%)
1	28 (30.8%)
Etiology	
Hepatitis B	86 (94.5%)
Others	5 (5.5%)
Child‐Pugh score	
A	81 (89.0%)
B	10 (11.0%)
AFP, ng/mL	
≥400	64 (70.3%)
<400	27 (29.7%)
Liver cirrhosis	
Yes	64 (70.3%)
No	27 (29.7%)
Tumor number	
1	24 (26.4%)
2	16 (17.6%)
≥3	51 (56.0%)
Tumor diameter, cm	
≥10	67 (73.6%)
<10	24 (26.4%)
Portal vein invasion	
Vp0	10 (11.0%)
VP3	35 (38.5%)
Vp4	46 (50.5%)
Extrahepatic spread	
Absent	75 (82.4%)
Present	16 (17.6%)
ALBI grade	
1	32 (35.2%)
2	59 (64.8%)

Abbreviations: AFP, a‐fetoprotein; ALBI grade, albumin‐bilirubin grade; ECOG PS, Eastern cooperative oncology group performance status; Vp, portal vein.

### 3.1 Efficacy

**FIGURE 2:**
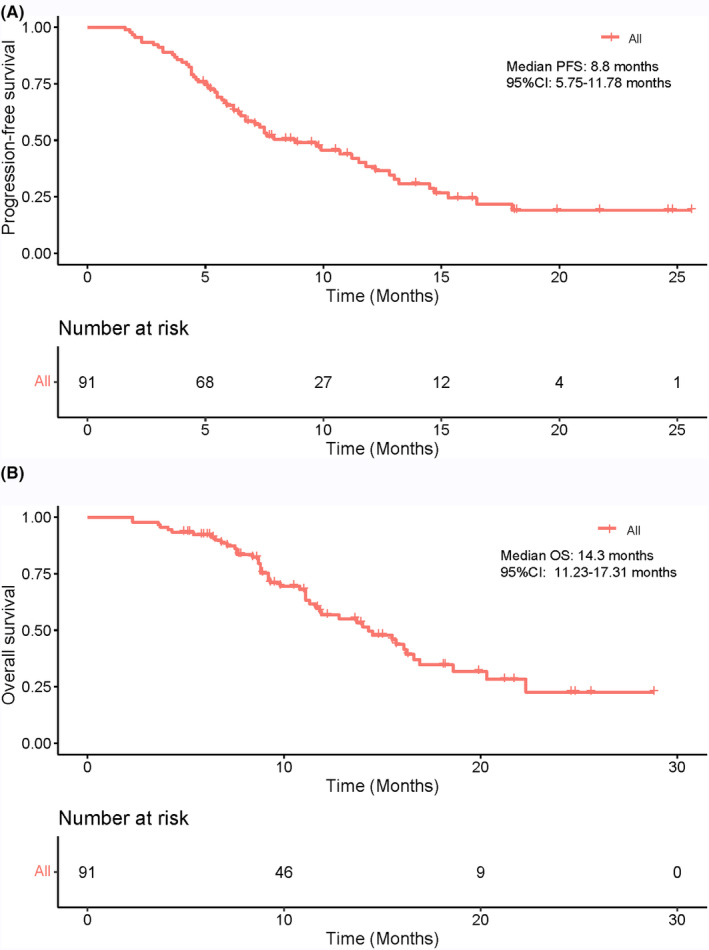
Kaplan–Meier curves for progression‐free survival and overall survival. OS, overall survival; PFS, progression‐free survival.

**FIGURE 3:**
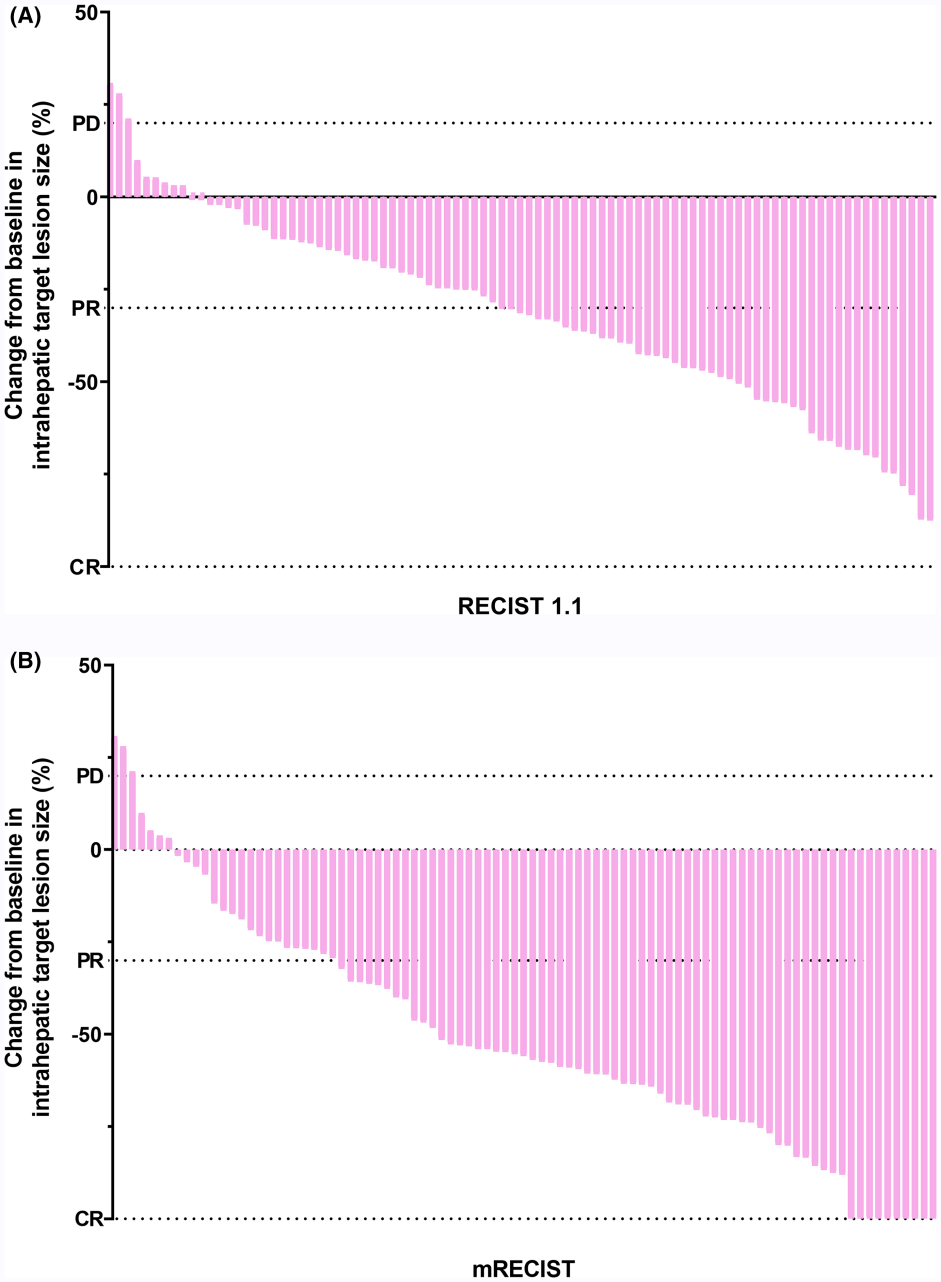
The best percentage change in size of intrahepatic target lesions relative to baseline in patients receiving HAIC combined with lenvatinib plus programmed cell death protein‐1 inhibitors; mRECIST: modified response evaluation criteria in solid tumors; RECIST, response evaluation criteria in solid tumors.

**FIGURE 4:**
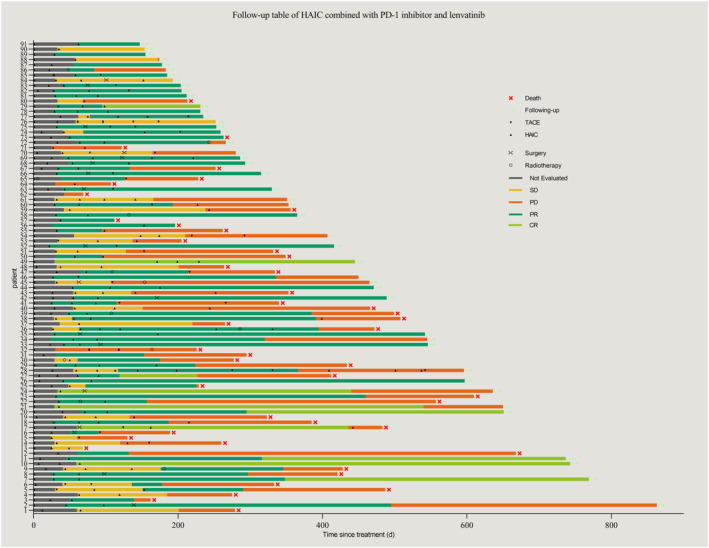
Treatments, survival, and tumor response of all patients, ranked by initial treatment time, evaluated by modified response evaluation criteria in solid tumors.

The data cutoff time was June 15, 2023, and the median follow‐up period was 16.3 months. As of the analysis, a total of 59 patients (64.8%) had experienced disease progression or mortality. The median PFS was 8.8 months (95% confidence interval [CI]: 5.75–11.78), as shown in Figure 2A, while the median OS was 14.3 months (95% CI: 11.23–17.31), as depicted in Figure 2B. During the study, a total of 285 HAIC cycles were administered, with a median of three cycles per patient. Tumor responses are summarized in Table 2. According to RECIST 1.1 criteria, none of the patients attained a CR. Out of the total number of patients, 48 (52.7%) attained PR, 39 (42.9%) had SD, and 4 (4.4%) experienced PD. The ORR was 52.7% and the DCR was 95.6%. According to mRECIST criteria, within the total patient cohort, 10 (11.0%) achieved CR, 56 (61.5%) attained PR, 21 (23.1%) had SD, and 4 (4.4%) experienced PD. The ORR was calculated to be 72.5%, and the DCR was 95.6%. Figure 3A,B illustrate the best change in the size of intrahepatic target lesions observed in the patients. The treatments, survival time, and tumor responses of all patients, ranked by the initial treatment time, are summarized in Figure 4. After the first‐line treatment progression, 38 patients continued to receive subsequent therapies. Details of the follow‐up treatments and the summary of patients undergoing local therapies are summarized in Table 3.

**TABLE 2:** Tumor responses.

	RECIST 1.1 (*n*, %)	mRECIST (*n*, %)
CR	0 (0%)	10 (11.0%)
PR	48 (52.7%)	56 (61.5%)
SD	39 (42.9%)	21 (23.1%)
PD	4 (4.4%)	4 (4.4%)
ORR	52.7%	72.5%
DCR	95.6%	95.6%

Abbreviations: CR, complete response; DCR, disease control rate; mRECIST: modified response evaluation criteria in solid tumors; ORR, objective response rate; PD, progressive disease; PR, partial response; RECIST, response evaluation criteria in solid tumors; SD, stable disease.

**TABLE 3:** Subsequent treatments.

Subsequent treatments	Number of patients
Second‐line treatment	
Regorafenib	8
Apatinib	4
Sintilimab + bevacizumab biosimilar	4
Regorafenib + PD‐1 inhibitors	10
Apatinib + PD‐1 inhibitors	7
Atezolizumab + bevacizumab	3
Tremelimumab + durvalumab	2
Local combination therapy	
Resection	18
Transarterial chemoembolization	30
Radiotherapy	13
Microwave ablation	2

Abbreviation: PD‐1 inhibitors, programmed cell death protein‐1 inhibitors.

### 3.2 Liver resection

**FIGURE 5:**
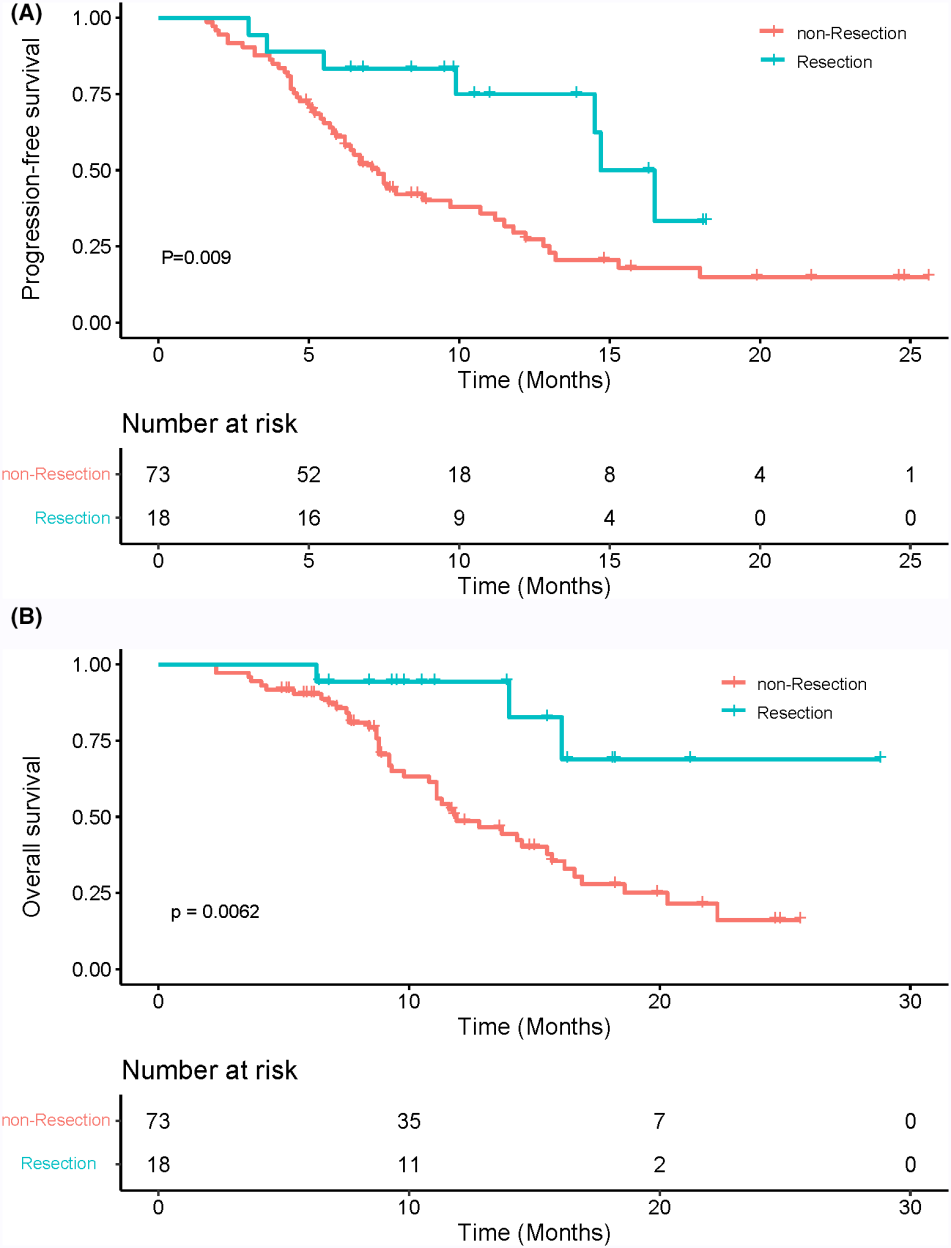
Kaplan–Meier curves for progression‐free survival and overall survival in the resection group and non‐resection group.

In our study, a total of 18 patients underwent surgical intervention, meeting the criteria for surgical resection and expressing willingness to undergo surgical treatment. A detailed summary of baseline characteristics for both groups can be found in the Supplementary Material. Significantly, differences were observed exclusively in the two groups prior to ALBI, with no discernible distinctions in other baseline indicators. Additional comprehensive details on the treatment plans, baseline information, and postoperative pathological status of the 18 surgically treated patients are provided in Supplementary Material. The Kaplan–Meier survival curves for PFS and OS in both the resection and non‐resection groups are presented in Figure 5 **(**Figure 5A,B **)**. Given the relatively short follow‐up duration, it is important to note that postoperative recurrence was not observed in over half of the patients in our cohort at the time of data collection.

### 3.3 Prognostic factor analysis

The survival prognostic factors are presented in Table 4. In the analysis of PFS, univariate analysis indicated significant associations between PFS and ECOG PS, liver cirrhosis, tumor number, and extrahepatic metastasis. Furthermore, in the multivariate analysis, ECOG PS 1 (*p* < 0.001; HR, 3.08; 95% CI 1.75–5.41), multiple tumors (*p* < 0.001; HR, 7.64; 95% CI 2.90–20.09), and extrahepatic metastasis (*p* < 0.027; HR, 2.21; 95% CI 1.09–4.48) were identified as a independent and significant factors associated with poor PFS. In terms of OS, univariate analysis demonstrated significant associations with sex, ECOG PS, Child‐Pugh score, tumor number, and extrahepatic metastasis. Subsequently, multivariate analysis identified child‐Pugh B (*p* = 0.020; HR, 2.52; 95% CI 1.16–5.52), multiple tumors (*p* < 0.002; HR, 26.73; 95% CI 3.22–222.07), and extrahepatic metastasis (*p* < 0.045; HR, 1.96; 95% CI 1.02–3.84) as a independent and significant factors associated with poor OS.

**TABLE 4:** Univariate and multivariate analysis of progression‐free survival and overall survival.

Variables	PFS				OS			
	Univariate cox regression		Multivariate cox regression		Univariate cox regression		Multivariate cox regression	
	HR (95% CI)	*p* value	HR (95% CI)	*p* value	HR (95% CI)	*p* value	HR (95% CI)	*p* value
Sex (male vs. female)	0.41 (0.16–1.05)	0.062			0.20 (0.05–0.83)	0.027	1.03 (0.23–4.69)	0.972
Age (<50 vs. ≥50)	0.81 (0.48–1.35)	0.418			1.02 (0.57–1.82)	0.958		
ECOG PS (0 vs. 1)	3.56 (2.11–6.01)	<0.001	3.08 (1.75–5.41)	<0.001	2.51 (1.40–4.48)	0.002	1.60 (0.87–2.96)	0.132
Etiology (others vs. hepatitis B)	1.06 (0.33–3.40)	0.928			1.86 (0.45–7.74)	0.395		
Child‐Pugh score (A vs. B)	2.66 (1.27–5.57)	0.009	1.64 (0.74–3.63)	0.220	3.82 (1.77–8.24)	0.001	2.52 (1.16–5.52)	0.020
AFP, ng/ml (<400 vs. ≥400)	1.38 (0.75–2.51)	0.299			1.00 (0.52–1.90)	0.997		
Liver cirrhosis (no vs. yes)	0.55 (0.32–0.94)	0.029	0.55 (0.30–1.02)	0.057	0.83 (0.45–1.52)	0.538		
Tumor number (single vs. multiple)	9.55 (3.72–24.56)	<0.001	7.64 (2.90–20.09)	<0.001	38.69 (5.24–285.81)	<0.001	26.73 (3.22–222.07)	0.002
Tumor diameter, cm (<10 vs. ≥10)	1.89 (1.00–3.57)	0.050			1.79 (0.88–3.62)	0.106		
Portal vein invasion (absent vs. present)	1.88 (0.68–5.20)	0.222			2.18 (0.53–9.04)	0.282		
Extrahepatic metastasis (absent vs. present)	3.78 (2.02–7.06)	<0.001	2.21 (1.09–4.48)	0.027	3.19 (1.66–6.11)	<0.001	1.96 (1.02–3.84)	0.045
ALBI grade (1 vs. 2)	1.61 (0.90–2.87)	0.109			1.78 (0.88–3.60)	0.109		

Abbreviations: AFP, a‐fetoprotein; ALBI grade, albumin‐bilirubin grade; CI, confidence interval; ECOG PS, Eastern cooperative oncology group performance status; HR, hazard ratio.

### 3.4 Safety

The frequencies of TRAEs have been tabulated in Table 5. Among all enrolled patients, 86 individuals (94.5%) experienced TRAEs, and no treatment‐related deaths occurred during the course of therapy. The most prevalent TRAEs encompassed proteinuria (50.5%), elevated aspartate aminotransferase (44.0%), abdominal pain (37.4%), hypertension (36.3%), and decreased appetite (33.0%). Notably, these adverse events were predominantly graded as 1–2 in severity. Furthermore, 26 patients (28.6%) encountered Grade 3–4 TRAEs, with the most common being hypertension (4.4%), thrombocytopenia (4.4%), elevated alanine transaminase (ALT) (4.4%), and esophageal variceal bleeding (4.4%). It is crucial to highlight that only one patient experienced Grade 4 hyperbilirubinemia, and their bilirubin levels gradually normalized posttreatment. Additionally, 4 patients (4.4%) had Grade 3 esophageal variceal bleeding, and timely endoscopic band ligation and hemostasis led to successful recovery. In terms of adverse events related to immunotherapy, we observed three (3.3%) severe immune‐related adverse events, including one case of Grade 3 immune‐related pneumonia and two cases of Grade 3 immune‐related hepatitis. For these patients, we implemented appropriate interventions, including the administration of corticosteroids or a combination of corticosteroids and immunosuppressants, resulting in symptom improvement. Additionally, during the arterial infusion of oxaliplatin, 36 (37.4%) patients experienced varying degrees of abdominal pain, including one patient who experienced Grade 3 abdominal pain. However, by reducing the infusion rate or temporarily discontinuing the administration of oxaliplatin and administering analgesics and lidocaine, the majority of patients rapidly obtained relief from pain.

**TABLE 5:** Treatment‐related adverse events.

Adverse event	Any grade (*n*, %)	Grades 1–2 (*n*, %)	Grade 3 (*n*, %)	Grade 4 (*n*, %)
Hypertension	33 (36.3)	29 (31.9)	4 (4.4)	0
Abdominal pain	34 (37.4)	33 (36.3)	1 (1.1)	0
Vomiting	23 (25.3)	23 (25.3)	0	0
Proteinuria	46 (50.5)	44 (48.4)	2 (2.2)	0
Thrombocytopenia	28 (30.8)	24 (26.4)	4 (4.4)	0
Hypothyroidism	25 (27.5)	24 (26.4)	1 (1.1)	0
Decreased appetite	30 (33.0)	30 (33.0)	0	0
Leukopenia	23 (25.3)	23 (25.3)	0	0
Hypoproteinemia	21 (23.1)	21 (23.1)	0	0
Hyperbilirubinemia	25 (27.5)	22 (24.2)	2 (2.2)	1 (1.1)
Hand–foot skin reaction	20 (22.0)	19 (20.9)	1 (1.1)	0
Elevated ALT	29 (31.9)	25 (27.5)	4 (4.4)	0
Elevated AST	40 (44.0)	38 (41.8)	2 (2.2)	0
Rash	5 (5.5)	4 (4.4)	1 (1.1)	0
Anemia	7 (7.7)	6 (6.6)	1 (1.1)	0
Diarrhea	17 (18.7)	17 (18.7)	0	0
Esophageal variceal hemorrhage	7 (7.7)	2 (2.2)	4 (4.4)	0
Immune‐related pneumonitis	2 (2.2)	1 (1.1)	1 (1.1)	0
Immune‐related hepatitis	5 (5.5)	3 (3.3)	2 (2.2)	0

Abbreviations: ALT, alanine aminotransferase; AST, aspartate aminotransferase.

## 4 DISCUSSION

The prognosis of HCC with high‐burden features (major PVTT [Vp3 and Vp4] and tumor infiltration of ≥50% of the liver) is extremely poor.^21^, ^22^ Recently, two phase II clinical trials have demonstrated that the combination of HAIC with systemic therapy can lead to improved prognostic outcomes.^6^, ^23^ Hence, we conducted this retrospective analysis to investigate the efficacy and safety of combining HAIC, lenvatinib, and PD‐1 inhibitors for high‐burden HCC in real‐world settings. Our study found that patients who received this combination achieved a median PFS of 8.8 months (95% CI, 5.75–11.78) and an OS of 14.3 months (95% CI, 11.23–17.31). Using RECIST 1.1 criteria, we observed an ORR of 52.7% and a DCR of 95.6%. Moreover, when assessed with mRECIST criteria, the ORR was 72.5%, with a corresponding DCR of 95.6%. These results suggest that triple therapy shows promise in delivering favorable therapeutic effects and enhancing survival outcomes for high‐burden HCC patients.

The results of the REFLECT trial showed that in terms of OS, lenvatinib is non‐inferior to sorafenib, with a median OS of 13.6 months.^14^ Consequently, lenvatinib has become the first‐line treatment for patients diagnosed with advanced HCC. In our study, according to the mRECIST criteria, 10 (11.0%) patients achieved a CR, with an ORR of 72.5% and a DCR of 95.6%. In contrast, the REFLECT trial reported only 6 (1%) patients in the lenvatinib group achieving a CR, with an ORR of 24.1% and a DCR of 75.5%. Regarding survival outcomes, our study showed a median OS of 14.3 months, similar to the REFLECT trial. However, it is worth noting that our study population may have a poorer prognosis as we exclusively included patients with tumor involvement of more than 50% of the liver volume and major PVTT (Vp3 and Vp4), while the REFLECT trial excluded patients with liver involvement of more than 50% and those with main PVTT. Recently, the combination therapy of atezolizumab and bevacizumab has emerged as a huge breakthrough in the systemic treatment of advanced HCC.^3^ Nonetheless, the updated IMbrave150 study revealed limited therapeutic benefits for patients with extensive liver involvement (more than 50% of the liver) and Vp4 portal vein tumor thrombus, with a median OS of just 7.6 months.^4^ In comparison, our study participants achieved a significantly higher OS compared to patients in the IMbrave150 trial who had Vp4 portal vein thrombus, bile duct invasion, or tumor involvement exceeding 50% of the liver volume. Although our study population differed from the subgroup in IMbrave150, there is evidence indicating poor prognosis in patients with major PVTT.^24^ A randomized Phase II trial comparing the effectiveness of combining HAIC with sorafenib versus sorafenib alone in patients with major PVTT showed slightly superior OS and PFS outcomes in the HAIC combination group compared to our combination therapy of HAIC with lenvatinib and PD‐1 inhibitors.^6^ In our study, multivariate analysis identified independent factors for PFS, including ECOG PS score, number of tumors, and presence of extrahepatic metastasis. Similarly, in the multivariate analysis of OS, independent risk factors were the Child‐Pugh score, number of tumors, and presence of extrahepatic metastasis. Compared to this Phase II trial, our patients had higher ECOG PS scores, a greater number of tumors, and more cases of extrahepatic metastasis. These factors might partially explain the persistently poor prognosis despite the addition of PD‐1 inhibitors. However, our study demonstrated a superior ORR and DCR according to both RECIST 1.1 and mRECIST criteria compared to the Phase II trial.

The observed survival benefits and excellent antitumor effects in our study can be attributed to a multitude of factors. First, the utilization of this triple combination therapy, which includes HAIC‐FOLFOX, lenvatinib, and PD‐1 inhibitors, holds the potential to elicit synergistic antitumor effects. On the one hand, HAIC can rapidly reduce tumor burden and improve patients' tolerance to systemic treatment, and thus prolong treatment duration.^6^ On the other hand, lenvatinib, a multitarget tyrosine kinase inhibitor (TKI), inhibits VEGFRs and FGFR, altering the tumor microenvironment (TME) and potentially enhancing the response to immunotherapy.^25^, ^26^, ^27^, ^28^ Additionally, oxaliplatin can induce immunogenic cell death also contributes to the enhanced response to immunotherapy.^29^ Second, in our study, a total of 18 patients underwent surgical resection, and Kaplan–Meier survival curves demonstrated a significant improvement in both PFS (*p* = 0.009) and OS (*p* = 0.006) in the resection group compared to the non‐resection group. Despite baseline differences in ALBI between the resection and non‐resection groups, Cox analysis, encompassing both univariate and multivariate analyses of PFS and OS, indicated that the impact of ALBI may not reach statistical significance. This result suggests that although there are some differences in ALBI between the surgical and nonsurgical groups, the influence may be relatively limited in survival analyses. Recent research suggests that the combination of TKI combined immunotherapy holds promise in increasing the likelihood of surgical resection for HCC patients initially deemed unsuitable for surgery.^30^, ^31^ These findings underscore the potential for transformative responses and conversion rates in unresectable HCC. Although prior studies have demonstrated poorer short‐term and long‐term outcomes for patients with major PVTT after surgical intervention, a retrospective study by Hamaoka et al. involving HCC patients with PVTT showed that among the 52 patients receiving combined HAIC and radiotherapy, 7 patients underwent surgical intervention. The study revealed a significantly improved overall survival in the resection group compared to the non‐resection group suggesting that HAIC‐based therapy might enhance the likelihood of curative resection, particularly in high‐burden patients.^32^, ^33^, ^34^

Regarding TRAEs, most of patients in our study experienced varying grades of TRAEs, which were generally manageable and did not result in TRAE‐related deaths. In contrast to the REFLECT study, we observed a higher occurrence of proteinuria, but the majority of cases were Grades 1–2 and did not significantly impact the treatment.^14^ Additionally, liver function abnormalities associated with TRAEs were also common but improved rapidly after completing HAIC and receiving hepatoprotective treatment. Abdominal pain was another common phenomenon, primarily occurring during arterial infusion of oxaliplatin. By reducing the infusion rate or temporarily stopping the administration of oxaliplatin, and providing analgesics and lidocain, the majority of patients experienced rapid pain relief. It is worth noting that during the treatment period, 4 patients experienced Grade 3 esophagogastric variceal bleeding, which may have been due to their pre‐existing severe liver cirrhosis. Although timely endoscopic variceal ligation effectively managed the bleeding, regular endoscopic examinations may be necessary for patients with severe liver cirrhosis to assess esophagogastric varices and intervene early. In our study, we observed three severe immune‐related adverse events, including one case of Grade 3 immune‐related pneumonia and two cases of Grade 3 immune‐related hepatitis. Among them, one patient with immune‐related pneumonia and one with immune‐related hepatitis showed symptom improvement after receiving intravenous methylprednisolone at a dose of 1 milligram per kilogram of body weight. However, for a patient with immune‐related hepatitis, despite receiving the same dose of methylprednisolone, liver function continued to deteriorate. Subsequent improvement was achieved after the addition of the immunosuppressant mycophenolate mofetil at a daily dose of 500 milligrams. In the current landscape of immunotherapy, vigilant monitoring of immune‐related adverse events is paramount, as these severe events can rapidly worsen, necessitating timely identification and appropriate intervention.

First, it is important to note that this is a retrospective study with a relatively small sample size and lacks a control group for comparison. Therefore, the possibility of selection bias cannot be ruled out. Second, it is essential to emphasize that in our study, the primary underlying cause among patients is infection with the hepatitis B virus (HBV), a distinctive feature not shared with regions such as the United States, Europe, and Japan.^35^ Concurrently, compared to global patient cohorts, Chinese patients manifest a higher incidence of microvascular invasion (MVI) and/or extrahepatic spread (EHS). Additionally, a greater proportion of Chinese patients are categorized into Barcelona clinic liver cancer (BCLC) stage C, indicating a more advanced clinical stage.^36^ Therefore, further research is required to explore the applicability of triple therapy for other types of hepatitis. Third, it is crucial to consider variations among PD‐1 inhibitors, as these could potentially impact patient responses and outcomes. Our center is currently conducting a unified protocol to further explore the efficacy of triple therapy. Lastly, considering the retrospective design of this study, there is a potential that adverse events may not have been thoroughly documented. Consequently, it is important to exercise caution when interpreting the findings of this study.

In conclusion, the utilization of HAIC‐FOLFOX in combination with lenvatinib and PD‐1 inhibitors as first‐line treatment shows significant promise for high‐burden HCC patients, with manageable adverse events. Thus, this combination therapy of HAIC, lenvatinib, and PD‐1 inhibitors represents a promising and innovative treatment option for high‐burden HCC. However, further prospective randomized controlled trials are necessary to validate above conclusions.

## AUTHOR CONTRIBUTIONS

Shumin Fu and Yongkang Xu made equal contributions to this work. **Shumin Fu:** Conceptualization (lead); data curation (lead); formal analysis (lead); methodology (lead); software (lead); writing – original draft (lead); writing – review and editing (lead). **Yongkang Xu:** Conceptualization (lead); data curation (lead); formal analysis (lead); methodology (lead); writing – original draft (lead); writing – review and editing (lead). **Wenjing Yan:** Data curation (equal); formal analysis (equal). **Ye Mao:** Project administration (equal); supervision (equal); writing – review and editing (equal). **Mengting He:** Data curation (equal); formal analysis (equal). **Zhimeng Chen:** Data curation (equal); formal analysis (equal). **Shenglan Huang:** Writing – review and editing (equal). **Dan Li:** Writing – review and editing (equal). **Yaqin Lv:** Writing – review and editing (equal). **Jianbing Wu:** Funding acquisition (lead); project administration (lead); supervision (lead); writing – review and editing (lead).

## FUNDING INFORMATION

This work was supported by the National Natural Science Foundation of China (No. 82060435), the Jiangxi Provincial Department of Science and Technology (No. 20202BAB206052), and the Clinical Research Project of the Second Affiliated Hospital of Nanchang University (2021efyC07).

## CONFLICT OF INTEREST STATEMENT

All authors have no conflicts of interest to declare.

## ETHICS STATEMENT

This study was approved by the Ethics Committee of the Second Affiliated Hospital of Nanchang University, China, with approval number: (2021) Medical Research Ethics Review (44‐01). Informed consent was obtained from each participant. Additionally, the trial has been registered with the Chinese Clinical Trial Registry, with registration number: Chic TR 2200064384.

## Supporting information


**Table 6.** Baseline characteristics of patients.

**Table 7.** Comprehensive profile of 18 surgical patients.

## Data Availability

Data supporting the results of this study can be obtained from the corresponding author, subject to a reasonable request.
