# Supplementary material for: Hepatic arterial infusion chemotherapy, lenvatinib plus programmed cell death protein‐1 inhibitors: A promising treatment approach for high‐burden hepatocellular carcinoma
Source: Cancer Med. 2024 Apr 30;13(9):e7105. doi: 10.1002/cam4.7105 (PMC11058683; doi:10.1002/cam4.7105)
Supplement: Supplementary file 1 — Table 6. Baseline characteristics of patients. [file CAM4-13-e7105-s002.docx]

**Table 6. Baseline Characteristics of Patients**

| **Characteristic** | **Non-resection groups**  **（n=73）** | **Resection groups**  **（n=18）** | **p** |
| --- | --- | --- | --- |
| Sex |  |  | 0.661 |
| Male | 66 (72.5%) | 15 (16.5%) |  |
| Female | 7 (7.7%) | 3 (3.3%) |  |
| Age, years | 51.4 (45.5, 58.0) | 47.8 (43.0, 55.3) | 0.218 |
| ≥50 | 41 (45.0%) | 10 (11.0%) |  |
| <50 | 32 (35.2%) | 8 (8.8%) |  |
| ECOG PS |  |  | 0.554 |
| 0 | 49 (53.8%) | 14 (15.4%) |  |
| 1 | 24 (26.4%) | 4 (4.4%) |  |
| Etiology |  |  | 1.000 |
| Hepatitis B | 69 (75.8%) | 17 (18.7%) |  |
| others | 4 (4.4%) | 1 (1.1%) |  |
| Child-Pugh score |  |  | 0.688 |
| A | 64 (70.3%) | 17 (18.7%) |  |
| B | 9 (9.9%) | 1 (1.1%) |  |
| AFP, ng/ml |  |  | 0.628 |
| ≥400 | 50 (54.9%) | 14 (15.4%) |  |
| <400 | 23 (25.3%) | 4 (4.4%) |  |
| Liver cirrhosis |  |  | 0.339 |
| Yes | 53 (58.2%) | 11 (12.1%) |  |
| No | 20 (22.0%) | 7 (7.7%) |  |
| Tumor number |  |  | 0.390 |
| 1 | 19 (20.9%) | 5(5.5%) |  |
| 2 | 11 (12.1%) | 5 (5.5%) |  |
| ≥3 | 43 (47.3%) | 8 (8.8%) |  |
| Tumor diameter，cm | 12.8 (9.6, 15.9) | 13.3 (10.5, 18.3) | 0.661 |
| ≥10 | 56 (61.5%) | 11 (21.1%) |  |
| <10 | 17 (18.7%) | 7 (7.7%) |  |
| Portal vein invasion |  |  | 0.999 |
| Vp0 | 8 (8.8%) | 2 (2.2%) |  |
| VP3 | 28 (30.8%) | 7 (7.7%) |  |
| Vp4 | 37 (40.7%) | 9 (9.9%) |  |
| Extrahepatic spread |  |  | 0.179 |
| Absent | 57 (62.6%) | 18 (19.8%) |  |
| Present | 16 (17.6%) | 0 (0%) |  |
| **ALBI grade** |  |  | 0.010 |
| 1 | 21 (23.1%) | 11 (12.1%) |  |
| 2 | 52 (57.1%) | 7 (7.7%) |  |

ECOG PS, Eastern Cooperative Oncology Group Performance Status; AFP, a-fetoprotein; Vp, portal vein; ALBI grade, Albumin-Bilirubin grade.
