# Supplementary material for: Hepatic arterial infusion chemotherapy, lenvatinib plus programmed cell death protein‐1 inhibitors: A promising treatment approach for high‐burden hepatocellular carcinoma
Source: Cancer Med. 2024 Apr 30;13(9):e7105. doi: 10.1002/cam4.7105 (PMC11058683; doi:10.1002/cam4.7105)
Supplement: Supplementary file 2 — Table 7. Comprehensive profile of 18 surgical patients. [file CAM4-13-e7105-s001.docx]

**Table7. Comprehensive Profile of 18 Surgical Patients**

|  | The types of PD-1 inhibitors | Time from surgery to initial treatment (months) | BCLC stage | Tumor number | Types of portal vein thrombosis | Intrahepatic  tumor size,  (cm) | Tumor  response, by  RECIST v1.1 | Tumor  response, by  mRECIST | Postoperative pathology status |
| --- | --- | --- | --- | --- | --- | --- | --- | --- | --- |
| 1 | Camrelizumab | 4.6 | C | ≥3 | 4 | 16.69 | PR | PR | pCR |
| 2 | Camrelizumab | 3.3 | C | 1 | 4 | 15.37 | PR | PR | pNR |
| 3 | Camrelizumab | 1.9 | B | ≥3 | 0 | 16.25 | PR | PR | pNR |
| 4 | Tislelizumab | 2.1 | C | 2 | 3 | 7.70 | PR | CR | pNR |
| 5 | Tislelizumab | 2.4 | C | 1 | 3 | 8.81 | PR | CR | pPR |
| 6 | Tislelizumab | 3.1 | C | 1 | 4 | 9.04 | PR | PR | pCR |
| 7 | Camrelizumab | 2.2 | A | 1 | 0 | 11.75 | PR | PR | pNR |
| 8 | Tislelizumab | 5.7 | C | 1 | 4 | 9.58 | PR | PR | pCR |
| 9 | Sintilimab | 2.1 | C | ≥3 | 3 | 11.39 | SD | SD | pPR |
| 10 | Sintilimab | 2.4 | C | 2 | 3 | 11.36 | PR | PR | pPR |
| 11 | Tislelizumab | 2.3 | C | 1 | 3 | 7.60 | SD | PR | pCR |
| 12 | Tislelizumab | 2.5 | C | 2 | 3 | 8.80 | PR | PR | pCR |
| 13 | Tislelizumab | 2.7 | C | 1 | 3 | 7.52 | PR | PR | pPR |
| 14 | Tislelizumab | 4.1 | C | 2 | 3 | 17.52 | PR | PR | pNR |
| 15 | Tislelizumab | 4.2 | C | 2 | 3 | 20.58 | SD | SD | pNR |
| 16 | Tislelizumab | 2.4 | A | 1 | 0 | 18.68 | PR | PR | pNR |
| 17 | Tislelizumab | 2.5 | A | 1 | 0 | 12.10 | SD | PR | pPR |
| 18 | Tislelizumab | 3.4 | C | 2 | 3 | 14.19 | SD | PR | pPR |

PD-1 inhibitors: programmed cell death protein-1 inhibitors; BCLC: Barcelona Clinic Liver Cancer; RECIST v1.1: Response Evaluation Criteria in Solid Tumors version 1.1; mRECIST; modified RECIST; pNR: Pathological Non-Response (> 50% viable tumor cells in the primary tumor or appearance of new lesions); pPR: Pathological Partial Response (≤50% viable tumor cells in the primary tumor); pCR: Pathological Complete Response (absence of viable tumor cells in resection specimens, including complete resection of primary tumors, tumor thrombosis, and lymph nodes).
